# Absolute Pitch: Effects of Timbre on Note-Naming Ability

**DOI:** 10.1371/journal.pone.0015449

**Published:** 2010-11-11

**Authors:** Patrícia Vanzella, E. Glenn Schellenberg

**Affiliations:** 1 Department of Music, University of Brasilia, Brasília, Brazil; 2 Department of Psychology, University of Toronto, Mississauga, Ontario, Canada; University of Oxford, United Kingdom

## Abstract

**Background:**

Absolute pitch (AP) is the ability to identify or produce isolated musical tones. It is evident primarily among individuals who started music lessons in early childhood. Because AP requires memory for specific pitches as well as learned associations with verbal labels (i.e., note names), it represents a unique opportunity to study interactions in memory between linguistic and nonlinguistic information. One untested hypothesis is that the pitch of voices may be difficult for AP possessors to identify. A musician's first instrument may also affect performance and extend the sensitive period for acquiring accurate AP.

**Methods/Principal Findings:**

A large sample of AP possessors was recruited on-line. Participants were required to identity test tones presented in four different timbres: piano, pure tone, natural (sung) voice, and synthesized voice. Note-naming accuracy was better for non-vocal (piano and pure tones) than for vocal (natural and synthesized voices) test tones. This difference could not be attributed solely to vibrato (pitch variation), which was more pronounced in the natural voice than in the synthesized voice. Although starting music lessons by age 7 was associated with enhanced note-naming accuracy, equivalent abilities were evident among listeners who started music lessons on piano at a later age.

**Conclusions/Significance:**

Because the human voice is inextricably linked to language and meaning, it may be processed automatically by voice-specific mechanisms that interfere with note naming among AP possessors. Lessons on piano or other fixed-pitch instruments appear to enhance AP abilities and to extend the sensitive period for exposure to music in order to develop accurate AP.

## Introduction

Absolute pitch (AP) is the ability to identify or produce a musical tone (e.g., middle C or concert A) without reference to an external standard [1]–[4]. AP requires memory for specific pitches, as well as links between mental representations of pitch and verbal labels (i.e., note names). It is a rare ability, with an estimated incidence below 0.01% in the general population [3]. Part of its rarity is due to the fact that AP can be evident only among those with music training; untrained individuals have no knowledge of note names. Indeed, when memory for the specific pitch of ecologically valid stimuli (e.g., pop songs, TV theme music, the dial tone) is examined without note-naming requirements, musically untrained children [5], [6] and adults [7]–[10] demonstrate remarkably accurate memory for pitch. Even young infants remember the pitch level of sung lullabies [11].

Normally, however, pitch is perceived relatively rather than absolutely by nonmusicians and musicians without AP. Relative pitch allows a listener to identify a familiar tune (e.g., *Yankee Doodle*) played at a novel pitch level, and to detect when a performer plays or sings a wrong note. Relative pitch is a more musically relevant mode of pitch processing than AP, which is actually associated with atypical development (e.g., autism [12], [13]; Williams syndrome [14]) and with non-human auditory processing [15], [16].

AP provides a unique opportunity to study interactions between linguistic and nonlinguistic information in memory for auditory stimuli, as well as related issues such as plasticity, sensitive periods, and genetics [17], [18]. In one survey of more than 600 musicians [19], the incidence of self-reported AP was 40% for individuals who started their music training at or before age 4, but only 3% for those starting at 9 years of age or later. In other words, not only does the development of AP depend on training, it also depends on training at an early age, typically by age 6 or 7 [20]. At the same time, early music training does not guarantee AP [19], [21], which suggests that AP requires a genetic predisposition in addition to early music lessons [19], [21]–[25].

There is also some agreement about AP on the following issues: (1) tone identification for AP possessors depends primarily on recognition of pitch chroma (i.e., note name: C, C-sharp, D, and so on) rather than pitch height (e.g., middle C vs C one octave higher) [26]–[28]; (2) response latency in note naming is a critical marker of AP, with AP possessors responding much faster than non-possessors, who may have memory for a single pitch and identify other notes based on pitch relations [27], [29]; (3) AP possessors tend to be better at identifying white-key compared to black-key notes, presumably because of greater familiarity [27], [30]; (4) compared to musicians without AP, AP possessors often perform *worse* on relative-pitch tasks [31]–[35]; (5) the prevalence of AP is higher among Asians than people with European backgrounds [21], [23], [36]–[38]; and (6) accuracy varies among AP possessors [30]. Although the distribution is sometimes considered to be bimodal (you have it or you don't) [39], note-naming ability among AP possessors actually ranges from excellent performance even with pure tones (i.e., sine waves, no overtones) to more moderate abilities that are sometimes enhanced for tones produced by a particular instrument, or *timbre* [26], [29].

Timbre is the attribute of tones that makes a middle C performed on a piano different from the same tone performed on a violin, even when both tones have the same duration and amplitude [40]. Except for a minority of individuals who are ‘infallible’ [26], AP possessors often perform differently depending on the timbre of the test stimuli. For example, AP possessors are better at identifying piano tones than pure tones [19], [28], [39], [41]. One comprehensive review concluded that singing is rarely used to evaluate AP performance because “of the difficulty of objectively determining the pitch of the sung tone” (p. 348) [3]. Although the authors offered two related explanations for the effects of timbre on AP performance, neither addressed the issue of difficulties with the human voice. One explanation was that AP possessors (and nonpossessors as well) appear to identify pitch more accurately on the first instrument they learned to play. The second was that greater familiarity with a timbre could make pitch identification easier. In line with this view, when violinists with AP are asked to tune a tone to concert A, performance is better for violin tones than for clarinet tones [42].

If familiarity and early experience are important predictors of good AP ability, why would the pitch of the human singing voice be difficult for AP possessors to identify? As noted by Belin et al. [43], “we probably spend more time everyday listening to voices than to any other sound, and our ability to analyze and categorize information contained in voices plays a key role in human social interactions” (p. 129). Thus, the question is raised of whether this difficulty actually exists, and if so, why. To the best of our knowledge, no study has examined AP possessors' performance with vocal tones compared with tones presented in other timbres. The present study provided such a comparison, contrasting performance among a large sample of AP possessors for natural sung tones with tones produced by a synthesized voice, piano tones, and pure tones. On the one hand, poorer performance with the natural voice compared to the synthesized voice would be easily attributable to *vibrato* (pitch fluctuations) in the natural voice, which would provide a simple explanation of the phenomenon if it exists. On the other hand, relatively poor performance for the natural *and* the synthesized voice compared to the nonvocal timbres would implicate a distinct status for processing voices.

Our interest in associations between timbre and AP also motivated us to test whether note-naming performance varied as a function of the particular musical instrument possessors first learned, and, thus, the timbre with which they first became familiar. We predicted that learning music on a fixed-pitch instrument such as the piano would enhance memory for pitch and increase note-naming accuracy. For fixed-pitch instruments, the pitch of specific tones does not change regularly as it does for variable-pitch instruments such as the violin. We also examined whether note-naming accuracy would be related to age of onset of music lessons, and whether this association might be moderated if the first instrument had fixed pitches. Although we know that AP possessors typically start lessons early in life, it is unclear whether individual differences in note-naming accuracy are also related to onset of music lessons.

Finally, our international sample allowed us to test whether training that involved fixed or moveable *do* influences note-naming abilities among AP possessors. Germanic and English-speaking countries tend to use a moveable *do*, such that *do* refers to the tonic (i.e., the most stable tone) of any scale (e.g., C in the key of C, C-sharp in the key of C-sharp, and so on). By contrast, Latin countries typically use a fixed *do* system, such that *do* always refers to pitch chroma C, and the tonic of scales other than C can be *re* (in the key of D), *mi* (in the key of E), and so on. One might speculate that AP performance would be enhanced among those trained with fixed *do*. Indeed, there is some evidence that AP is more prevalent among US music students who have experience with a fixed-*do* system (e.g., the Yamaha method) [21]. Again, this finding does not speak to individual differences among AP possessors. Moreover, note names (C, C-sharp, and so on) are used frequently in moveable-*do* systems and these names always refer to the same tones. In other words, the difference between the two systems may be irrelevant for AP performance.

## Methods

### Participants

The present study was approved by the Office of Research Ethics at the University of Toronto. Because the study was conducted on-line, all participants were informed at the beginning of the study that by completing the test they were consenting to participate. Participants were recruited by word of mouth, primarily through contacts made by the first author (a professional pianist and music professor). Other participants were recruited through on-line lists that cater to researchers interested in audition or music cognition. Recruitment efforts specified that participants should have AP or at least suspect that they have AP. Our test was completed 323 times but several people did the test more than once. Data from only the first test session for each participant were retained for analysis. We also excluded people who gave obviously fictitious answers to the demographic questions.

We determined statistically whether each participant exhibited evidence of AP by comparing performance for each of the four timbres with chance levels, correcting for multiple tests. Some researchers analyze AP scores twice, considering semitone errors as correct or incorrect [31]. Others consider semitone errors to be correct for participants 45 years of age or older, and to be worth ¾ of a point for other participants [39]. Still others award ¾ of a point for semitone errors for all participants [34]. Because our sample was, on average, older than others, we chose to use a more liberal criteria and counted semitone errors as correct for all participants (as in [24], [44]). Regardless, when determining whether an individual exhibits evidence of possessing AP, it is irrelevant whether semitone errors are considered correct because chance-level responding changes accordingly. Moreover, awarding ¾ of a point or a full point for semitone errors was a moot point in the statistical analyses because the two scoring methods were almost perfectly correlated (*r*s>.98) for each of the four timbres.

Because semitone errors were considered as correct, there were 3 correct responses out of 12 options on each trial. Thus, chance performance on 24 trials for each timbre was 6 correct, and participants required a score of 11 or greater to be significantly better than chance. Using the normal approximation to the binomial (without correcting for continuity), a score of 11 results in a *z*-statistic of 2.36 and a corresponding *p*-value of .009 (one-tailed; below-chance performance was uninterpretable). The final sample comprised participants who performed better than chance on at least one of our four timbres. Defining AP as performing better-than-chance on a note-naming task was a liberal criterion but our goal was to examine variation in AP accuracy. Accordingly, it was important that the range of ability was not restricted to the most accurate individuals.

Our final sample of 198 participants included approximately equal numbers of men (*n* = 107) and women (*n* = 91). The mean age was 30 years (*SD* = 12) and the median was 26 years. The most common country of residence was Brazil (43%) but many participants lived in the US (22%), Europe (18%), or Canada (12%). When participants were asked whether they had AP prior to beginning the test session, 56% responded *yes*, 40% responded *I don't know*, and 5% responded *no*. In other words, almost half of the sample exhibited evidence of having AP without overt knowledge of their AP status. As one would predict from previous reports, most of our AP possessors began music lessons as young children. Cumulative frequencies indicated that 35%, 51%, 63%, and 71% began by age 5, 6, 7, and 8, respectively. Nevertheless, 9% of participants did not begin lessons until they were teenagers or older, and one participant started lessons at age 46. These results are consistent with a gamma distribution for age of onset of music lessons among AP possessors [45].

Approximately half of the participants (51%) reported that their first instrument was piano, whereas violin or viola were listed as the first instrument by 21%. Other first instruments (e.g., cello, guitar, recorder) were listed less frequently. Questions about current musical activities (not mutually exclusive) revealed that 40% of participants were music students, 34% were professional musicians, 29% were music teachers, 22% were amateur musicians, and 9% were professors or instructors in music at a university or college; 16% and 11% reported having had extensive or some training in music, respectively, but that they no longer played on a regular basis.

### Stimuli and apparatus

The stimuli were 96 1-s digital samples, 24 in each of four timbres, which included all notes from the chromatic scale between A3 (220 Hz, 3 semitones below middle C) and G-sharp5 (831 Hz, 20 semitones above middle C). The tones were in equal-tempered tuning with the fundamental frequency of A4 (concert A, the A above middle C) set to 440 Hz (standard tuning). All 96 stimuli were normalized so that the maximum amplitude was identical. Although perceived loudness may have varied as a function of pitch height, differences in loudness would have been distributed similarly for each of the four timbres.

The piano and synthesized-voice stimuli were generated originally on a Roland FP-4 keyboard using the Grand Piano 1 and GM2 Voice timbres, respectively. They were digitally edited using SoundEdit to be exactly 1 s, with a natural onset and a 10-ms linear offset. The sine waves were created in SoundEdit and included 10-ms linear onsets and offsets. The natural vocal stimuli were sung by a professional soprano (Adélia Issa) and recorded in a professional recording studio (Estúdio Mickael) in São Paulo. The singer heard each tone played on a piano and then sang the same pitch with the vowel/a/into a Neumann U67 microphone. She sang each of the 24 tones repeatedly until she and the experimenter were satisfied with her performance. The singer was encouraged to minimize her vibrato while maintaining her natural singing voice. Her vocal productions were saved as digital sound files with a high signal-to-noise ratio using ProTools. A 1-s portion of each sound file was selected subsequently using SoundEdit, such that the amplitude of the stimulus was as constant as possible, and any vibrato was confined primarily to the second half. Each sung stimulus tone was then ramped with 10-ms linear onsets and offsets.

We used Praat software [46] to analyze the amount of pitch variation in the middle (steady state) portion of each test tone (from 250 to 750 ms). The fundamental frequency was calculated in 10-ms intervals and the standard deviation for each test tone was recorded in semitones. The pure tones had no variation in pitch (Praat reported a mean *SD*<1/100000 of a semitone, averaged across the 24 test tones), whereas the piano (mean *SD*<1/100 of a semitone) and synthesized vocal (mean *SD*<1/20 of a semitone) tones had minor variations. By contrast, the natural vocal tones had a substantial amount of variation, with a mean standard deviation greater than 1/5 of a semitone. An Analysis of Variance (ANOVA) was conducted to determine whether the standard deviation in pitch varied across timbres, treating the test tone as the experimental unit. The analysis confirmed that standard deviations did indeed differ, *F*(3, 92) = 59.09, *p*<.0001, η^2^ = .658. The natural vocal tones had more variation in pitch compared to the other three timbres, *p*s<.0001 (Tukey HSD). There were no differences among the three sets of computer-generated test tones (piano, pure tones, synthesized voice), *p*s>.3, however, because in each instance they had a relatively consistent pitch.

### Procedure

Testing was conducted on-line (http://perfectpitch.freehostia.com/) using a customized software program created with Adobe Flash. The program was modeled after the one used by Athos et al. [39]. It was available in either English or Portuguese. It first required participants to answer demographic questions about their age and sex as well as their musical background, their current musical activities, and their experience of having AP, including their subjective impressions about particularly difficult timbres for note naming. Participants were then instructed to adjust the volume of their computer speakers to a comfortable level while they listened to a non-diatonic sequence of ascending square-wave tones.

The actual test session comprised four blocks, one for each timbre, with blocks presented in random order. The order of the 24 test tones within each block was also randomized. Within blocks, test tones were separated by only 3 s, which effectively precluded using relative pitch as a response strategy [27], [29]. During this window, participants responded by clicking one key on a display of 12 keys that looked like a piano keyboard. Each key was labeled with its note name, with black keys having two labels (e.g., C-sharp and D-flat). Because a fixed-*do* system is used in Brazil, the labels in the Portuguese version of the program were in *solfege* with Portuguese spellings. Participants were allowed to take breaks between but not within blocks. After they completed the fourth and final block, their scores on each of the four blocks were presented. No feedback was provided beforehand.

## Results

**Figure 1:**
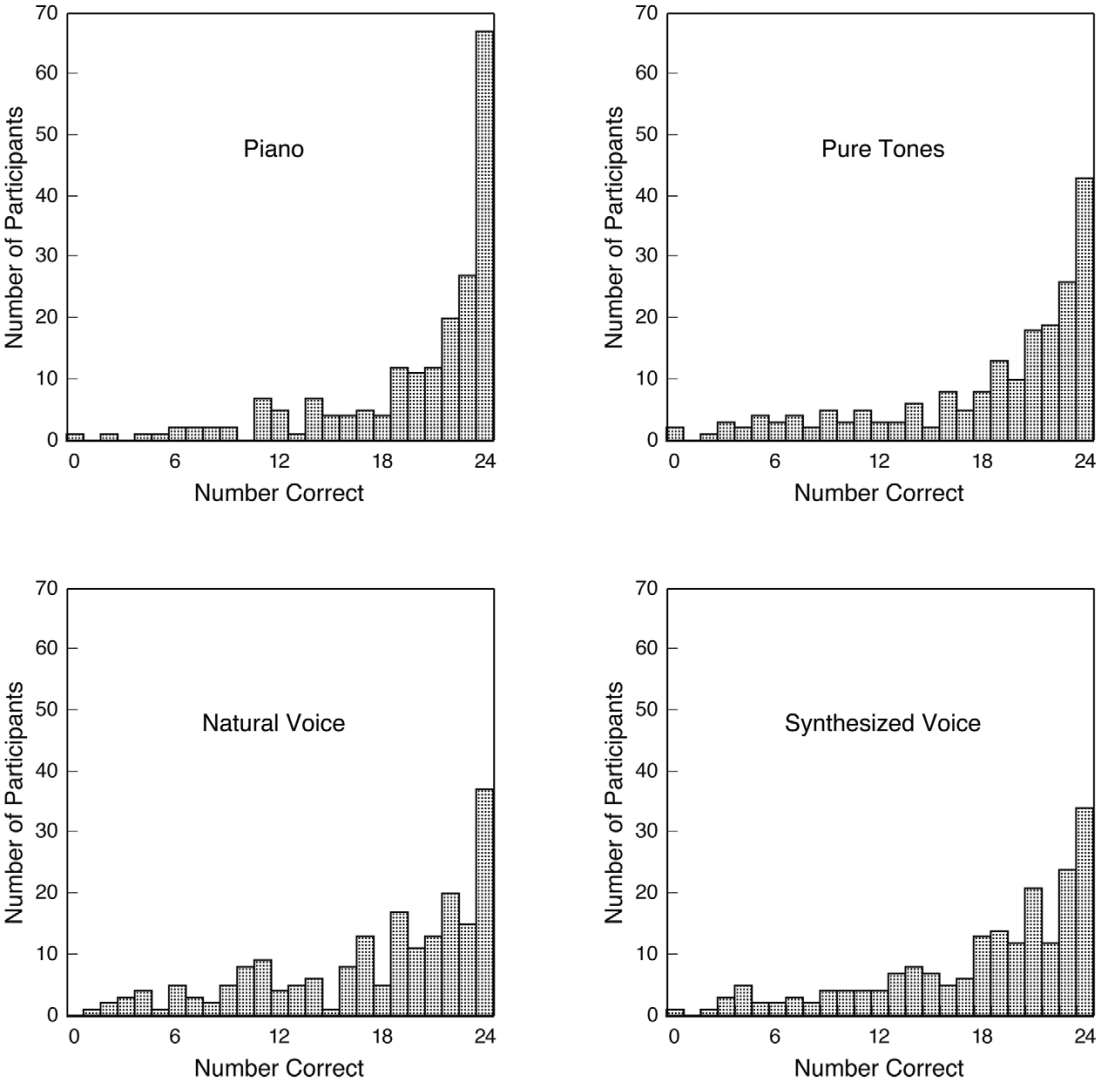
Histograms illustrating performance separately for each of the four timbres. Although the mode was perfect performance for each timbre (semitone errors counted as correct), the figure illustrates substantial individual differences in performance in each instance.

Histograms in Figure 1 illustrate performance separately for each of the four timbres. Although the mode was 100% correct in each case (with semitone errors counted as correct), for each timbre the vast majority of participants made some errors. Indeed, on each timbre some participants scored rather poorly (i.e., at or even below chance levels), indicating marked individual differences in AP performance. Participants who scored relatively high (or low) on one timbre tended to score high (or low) on the other timbres as well, .75≤*r*s≤.81, *N* = 198, *p*s<.0001.

The principal analysis was a one-way repeated-measures ANOVA with timbre as the independent variable. Performance varied reliably across timbres, *F*(3, 591) = 31.42, *p*<.0001, η_p_^2^ = .138. Planned comparisons confirmed that, as in previous research [19], [28], [39], [41], performance was better for piano tones (*M* = 84%, *SD* = 22%) than for pure tones (*M* = 77%, *SD* = 26%), *t*(197) = 5.95, *p*<.0001, η_p_^2^ = .152. Performance did not vary significantly between the natural (*M* = 73%, *SD* = 26%) and synthesized (*M* = 75%, *SD* = 25%) voices, *p*>.05, however, which indicates that vibrato did not influence performance to a notable degree. As expected, performance averaged across the two vocal timbres (*M* = 74%, *SD* = 24%) was significantly worse than it was with piano tones, *t*(197) = 9.43, *p*<.0001, η_p_^2^ = .311. More surprising, however, was the finding that performance on the vocal timbres was significantly worse than performance with pure tones, *t*(197) = 2.94, *p*<.005, η_p_^2^ = .042. Identical response patterns emerged when semitone errors were not considered to be correct, and when we analyzed relatively good and poor performers separately (i.e., using a median split based on overall performance). Additional pairwise comparisons revealed that the difference between pure tones and the natural voice was significant, *p* = .001, whereas the difference between pure tones and the synthesized voice was marginal, *p* = .1. When semitone errors were not considered to be correct, both comparisons were significant favoring pure tones, *p*s<.05.

Overall performance (summed across the four timbres) was used to examine the association between note-naming ability and the age when participants started music lessons (one participant who reported beginning music lessons at age 1 was excluded from these analyses). The correlation was negative, *r* = −.11, *N* = 197, but not statistically significant, *p*>.1. Nonetheless, there could still be a critical or sensitive period for enhanced note-naming performance among AP possessors, as there is for exhibiting AP in general [20]. To identify the appropriate cut-off point, we compared those with and without early music lessons four times, using the entire sample in each analysis but changing the definition of ‘early’ in each analysis (i.e., by age 5, 6, 7, or 8). Participants who began lessons by age 5 (*M* = 82%, *SD* = 20%) scored significantly higher than other participants (*M* = 74%, *SD* = 23%), *t*(195) = 2.24, *p*<.05, η^2^ = .025, as did participants who began by age 6 (*M* = 82%, *SD* = 20% vs *M* = 72%, *SD = *24%), *t*(195) = 3.33, *p* = .001, η^2^ = .054. The strength of the association peaked when the cut-off was set at beginning lessons by age 7 (*M* = 81%, *SD* = 20% vs *M* = 70%, *SD* = 25%), *t*(195) = 3.37, *p*<.001, η^2^ = .055. When the cutoff was set at age 8 or higher the difference in performance between those with or without early music lessons was no longer significant, *p*>.05. Accordingly, beginning lessons by age 7 was used as the criterion in subsequent analyses that compared participants with (*n* = 124) and without (*n* = 73) early music lessons. We re-ran the original ANOVA on differences among timbres including early music lessons as a between-subjects variable. There was no interaction between timbre and early lessons, *p*>.3. In short, early music training was predictive of better performance across the four timbres.

The next analysis compared performance between participants whose first instrument was the piano (a fixed pitch instrument, *n* = 101) and those who began their training on other instruments (typically without fixed pitches, *n* = 97). In line with our hypothesis, overall performance was higher for the piano group (*M* = 82%, *SD* = 19%) compared to their counterparts (*M* = 72%, *SD* = 25%), *t*(196) = 3.31, *p* = .001, η^2^ = .053. We re-ran the initial ANOVA on differences among the four timbres including first instrument as a between-subjects variable. There was no hint of a two-way interaction, *F*<1. Thus, the advantage for the piano group extended across the four timbres. A parallel analysis compared participants whose *current* regular instrument was piano (*n* = 75) with all other participants (*n* = 123). There was no main effect of current piano playing, *p*>.1, and no interaction with the timbre manipulation, *F*<1.

**Figure 2:**
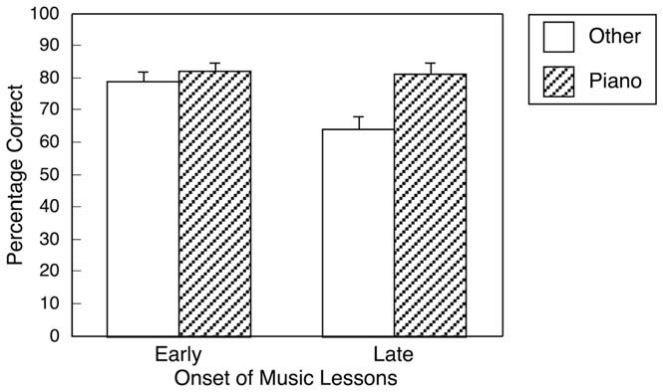
Performance as a function of age when music lessons began and the first instrument learned. Performance was summed across the four timbres. Early music lessons are those that started by 7 years of age. Error bars are standard errors. The figure illustrates improved performance for those who started music lessons on piano *or* at an early age, but no additional improvement for those who started music lessons on piano *and* at an early age.

Participants who started music lessons relatively late in life (i.e., at age 8 or later) were more likely than other participants to have piano as their first instrument, χ^2^(1, *N* = 197) = 11.37, *p*<.001 (odds ratio  = 2.77). A multiple regression model with two dummy-coded predictor variables (early lessons, first instrument) accounted for 9% of the variance in overall performance, *F*(2, 194) = 9.44, *p* = .0001. Both predictor variables made significant unique contributions to the model's explanatory power that were almost identical in magnitude (early lessons: *sr^2^* = .035, first instrument: *sr^2^* = .033, *p*s<.01). When the two-way interaction term was added to the model, it significantly improved explanatory power by 2%, *F*_inc_(1, 193) = 4.31, *p*<.05. Figure 2 illustrates overall performance as a function of early lessons and first instrument. Follow-up analyses indicated that for those who started music lessons early in life, first instrument was independent of overall performance, *p*>.3. By contrast, for those who started lessons after age 7, performance was enhanced among those who started on the piano, *t*(71) = 2.97, *p*<.005, η^2^ = .110. As shown in Figure 2, performance was better when *either* variable was present (early lessons *or* piano as first instrument) compared to when neither variable was present, whereas the presence of both variables was not associated with further improvement.

Participants were also classified based on whether their music training used a system with fixed or movable *do*. The fixed-*do* group comprised participants who were nationals *and* current residents of a Latin country (e.g., Brazil, France, Italy, Portugal, Spain; *n* = 86), whereas the movable-*do* group comprised participants who were nationals *and* current residents of a Germanic or English-speaking country (e.g., Canada, Germany, Netherlands, UK, US; *n* = 79). Participants with a mixed background (e.g., a Brazilian national currently living in the US) were excluded from the analysis. The fixed- and movable-*do* groups did not differ in note-naming ability, *p*>.3. In fact, the movable-*do* group (*M* = 79%, *SD* = 25%) performed slightly better than the fixed-*do* group (*M* = 75%, *SD* = 20%).

The final set of analyses examined self-reports that participants found the pitch of particular timbres difficult to identify, specifically *voices* for some participants, and *electronic and synthesized sounds* for others. (Self-reported difficulties with other timbres were too infrequent to allow for meaningful comparisons.) These analyses were important because participants were not tested in the laboratory and they may have modified their performance systematically to fit their hypotheses about the study. Individuals who claimed that they had difficulty identifying the pitch of voices (*n* = 77) did not differ from other participants (*n* = 121) on their actual pitch-naming performance in the natural voice (*M* = 71%, *SD* = 24% vs *M* = 74%, *SD* = 27%) or synthesized voice (*M* = 72%, *SD* = 23% vs *M* = 77%, *SD* = 26%) condition, *p*s>.2. These null findings suggest that participant bias (or demand characteristics) did not play an important role in the observed difficulty participants had with the vocal timbres. Moreover, participants who claimed that they had difficulty identifying the pitch of electronic and synthesized sounds (*n* = 67) performed similarly to other participants (*n* = 131) in the pure-tone (*M* = 75%, *SD* = 26% vs *M* = 78%, *SD* = 26%) and synthesized-voice (*M* = 73%, *SD* = 22% vs *M* = 76%, *SD* = 26%) conditions, *p*s>.3. In short, we found no evidence that participants attempted to modify their actual performance on our note-naming task to be consistent with their claims about difficulties identifying the pitch of certain timbres.

## Discussion

To the best of our knowledge, only one study [39] tested a sample of AP possessors larger than ours. As in the present study, the authors recruited their participants over the internet. Whereas their focus was on differences between AP possessors and nonpossessors, ours was on variation in note-naming ability among individuals with AP. Although internet studies are less controlled than laboratory studies, they afford a unique opportunity to recruit a large sample from a rare population. Moreover, we found no evidence that participants modified their performance to conform with their beliefs about AP.

AP possessors had more difficulty identifying the pitch of vocal tones (natural or synthesized) than they did with nonvocal tones (piano or pure tones). Our findings also indicated that possessors' difficulty at identifying the pitch of voices cannot be attributed solely to vibrato, which was much more pronounced in the natural compared to the synthesized vocal stimuli. In fact, vibrato in the synthesized vocal tones was not significantly greater than it was in the piano or pure tones.

One explanation for our finding of note-naming difficulty for voices among AP possessors is provided by studies of the neural correlates of voice perception [43], [47], [48]. The human voice carries a wealth of paralinguistic information, including affective and identity cues, which are conveyed simultaneously in speech along with linguistic information. Specific brain regions and mechanisms appear to be involved in processing the human voice because of its inherent link with linguistic *and* paralinguistic information [47], and these regions and mechanisms could be distinct from those used with non-referential auditory stimuli.

In line with this view, when participants listen passively to vocal or nonvocal stimuli, functional magnetic resonance imaging identifies “voice-sensitive cortical regions” that exhibit greater levels of brain activity in response to vocal sounds [43]. When neural responses to sung voices or musical instruments are compared, cortical activation is stronger for the vocal stimuli [49], [50]. Brain-activation responses to human voices are also more rapid than responses to bird songs or environmental sounds [51]. Belin et al. [43] hypothesized that the mechanism of voice recognition could thus be similar to that of face perception, with linguistic, affective, and identity information processed simultaneously and automatically in distinct but non-independent cortical pathways. Decoding meaning from prosodic and lexical information in speech may be particularly rapid and automatic among AP possessors [52] because of distinctive neural pathways that are necessary for speech and music processing [53]. For atypically developing AP possessors, however, such as those with autism who have marked deficits in social functioning, interference from the human voice may be negligible. In one case study, a high-functioning autistic individual with AP was better at identifying the pitch of speech compared to non-autistic AP possessors [54].

Behavioral studies provide additional evidence for the close coupling of linguistic and paralinguistic information in voice perception, whether the voice is spoken or sung. For example, when adult [55] and child [56] listeners are required to identify whether they previously heard a spoken word, performance is less accurate when the talker changes from the exposure to the test phase. In other words, listeners cannot completely ignore paralinguistic information that is irrelevant to the task. Similar results from the elderly provide converging evidence that linguistic and paralinguistic (i.e., indexical or talker-specific) cues are processed in tandem [57].

We speculate that hearing voices or voice-like stimuli automatically activates neural pathways devoted to decoding linguistic *and* paralinguistic information, and that this activation interferes with the identification of the pitch of stimuli produced by the human voice. More specifically, for AP possessors, voices are inextricably linked with decoding *meaning*, which interferes with decoding non-referential information (pitch chroma), particularly when the task also requires mappings with atypical linguistic information (i.e., note names). This interpretation is consistent with proposals that the rarity of AP stems from difficulty associating names with isolated pitches rather than from individual differences in pitch memory [8], [9], [58]. Greater variability among singers compared to instrumental performers in targeting and maintaining specific pitches may also play a role in the decrements with voices reported here. Nonetheless, because these difficulties generalized to a professional but unfamiliar opera singer as well as to a synthesized vocal timbre with little vibrato, this interpretation is still consistent with the proposal that human voices represent a special class of auditory stimuli.

We also found that note naming was better among participants who began music lessons earlier in life (by age 7), and among those who began their training on piano, and that these two factors interacted in their influence on performance. Whereas piano training enhanced performance of those who started music lessons at a relatively late age, there was no such effect for children who started lessons at a younger age. Indeed, *either* factor (early lessons, starting on piano) improved note-naming performance equally, and the presence of *both* factors did not improve performance further. Strictly speaking, our quasi-experimental design precludes inferences of causation. Nonetheless, although a predisposition for accurate AP may increase the likelihood of taking music lessons early in life, it seems unlikely that such a predisposition would increase the likelihood of starting music training specifically on the piano later in childhood. Rather, the available evidence points to a sensitive period for exposure to music lessons in order to exhibit AP [20]. Our results are important for uncovering a sensitive period for developing accurate AP ability, and for demonstrating that this sensitive period may be extended in time if piano is the first instrument studied. We attribute this finding to the fact that—unlike voices and violins—pianos have fixed pitches. Presumably, hearing a note such as middle C at exactly the same pitch from day to day would promote a more stable mental representation of its pitch.

Because piano tones are frequently used as stimuli in tests of AP ability, and because piano is the most common musical instrument, some scholars [4] have suggested that researchers often measure ‘absolute piano’ rather than true AP ability. We found no evidence of enhanced AP for piano tones among piano players. Rather, piano tones were the easiest to identify among *all* participants, and piano training was a predictor of good performance *across* our four stimulus timbres, but there was no evidence of a special link between piano training and piano tone identification. Our hypothesis about fixed pitches could be tested in future research by recruiting AP possessors who began lessons on fixed-pitch instruments other than piano, such as organ or vibraphone. We predict that compared to their counterparts trained on instruments without fixed pitches, these participants should exhibit enhanced note-naming ability regardless of the test timbre. Although familiarity with a particular instrument is implicated in previous findings of an advantage for violin over clarinet tones among violinists [42], the advantage may be restricted to comparisons between two variable-pitch instruments or to the particular task, which involved tuning the pitch of a tone to concert A.

Whereas Gregersen et al. [21] reported an increased likelihood of AP among US music students who had some training on a fixed-*do* musical system, we found no evidence of a difference in note-naming ability between participants who came from Latin countries (with fixed *do*) and other participants. Even if a small proportion of our sample were trained on systems atypical for their country (e.g., US participants who studied with the Yamaha method), our large sample size provided us with sufficient power with this added noise in the data. Moreover, our participants from moveable-*do* countries actually performed slightly better than participants from fixed-*do* countries. One possible explanation is that training on a fixed-*do* system increases the likelihood of exhibiting AP without leading to individual differences in note-naming performance among AP possessors. It is also possible that Grergersen et al.’s participants with fixed-*do* training actually had *more* or *earlier* music training than their counterparts, or that the finding was a Type I error. Indeed, their sample included 130 participants from the US with self-reported AP, such that the number with training on a fixed-*do* system (not reported) was bound to be very small. In short, it remains an open question whether training with fixed *solfege* names (fixed *do*) or fixed letter names (moveable *do*) influences the development of AP.

In sum, we observed that the identification of sung tones (natural or synthesized) is difficult for AP possessors, perhaps due to the simultaneous linguistic and paralinguistic information typically carried by the human voice. From this view, the link between voices and meaning interferes with decoding non-referential information that must, at the same time, be associated with a verbal label (i.e., a note name). Our results also provided evidence that in order to develop the most accurate note-naming abilities, a child typically needs to start music lessons by age 7. This sensitive period may be extended, however, if music lessons are begun on a piano or another fixed-pitch instrument.
